# Fabrication and Characterization of a Multichannel 3D Thermopile for Chip Calorimeter Applications

**DOI:** 10.3390/s150203351

**Published:** 2015-02-03

**Authors:** Tho Phuoc Huynh, Yilei Zhang, Cohen Yehuda

**Affiliations:** 1 School of Mechanical and Aerospace Engineering, Nanyang Technological University, 50 Nanyang Avenue 639798, Singapore; E-Mail: phuoctho001@e.ntu.edu.sg; 2 Singapore Centre on Environmental Life Sciences and Engineering, Interdisciplinary Graduate School, Nanyang Technological University, 60 Nanyang Drive 637551, Singapore; E-Mail: ycohen@ntu.edu.sg

**Keywords:** chip calorimeter, microfabrication, 3D thermopile

## Abstract

Thermal sensors based on thermopiles are some of the most robust and popular temperature sensing technologies across industries and research disciplines. A chip calorimeter with a 3D thermopile layout with a large sensing area and multichannel capacity has been developed, which is highly desired for many applications requiring large reaction chambers or high throughputs, such as biofilm research, drug screening, *etc.* The performance of the device, including temperature sensitivity and heat power sensitivity, was evaluated. The capability to split the chip calorimeter to multiple channels was also demonstrated, which makes the chip calorimeter very flexible and powerful in many applications.

## 1. Introduction

Thermal sensors based on thermopiles are some of the most robust and popular temperature sensing technologies across industries and research disciplines. Using the integrated circuit (IC) fabrication technology, thermopiles which consist of thermocouples connected in series could nowadays be fabricated on the micrometer scale [1,2]. The microfabricated chip calorimeter uses a thermocouple array (thermopile) to detect the heat involved in a chemical or biological process, such as the development of bacterial biofilms on surfaces [3,4], the response of biofilms to various antibiotics and predation agents [5–7], thermal transition and heat of mixing of substances [8], growth of single zebra fish embryos [9], DNA hybridization [10], heat transfer from the activity of enzymes [11], heat flow from single or a few fat cells [12]. A commercially available chip calorimeter were also designed and marketed [3,13,14].

Usually those thermocouples with a few micrometers in width are laid on silicon wafer surfaces by consecutive thin film deposition and etching. Bulk silicon is selectively removed by alkaline back etching to release free standing SiO_2_/Si_3_N_4_ membranes to serve as the hot junctions. The samples to be measured using the chip calorimeter could be in either an open chamber or a closed chamber. If a small and open chamber is used, the liquid sample to be measured is subjected to high evaporation rate; thus, a closed chamber is usually made and attached to the device for flow experiments [14,15]. Furthermore, those free-standing thermopiles are very fragile and can easily be broken during experiments.

Another limitation to the applications of chip calorimeters is due to the in-plane thermocouple layout [4], *i.e.*, all hot and cold junctions have to share the same plane at the top of the device, which means that part of the top surface is used for the cold junctions instead of the hot junctions, or the sensing area. In the past decade, 3D thermopiles with vertical thermocouples were developed [16–20]. Instead of placing all the junctions on the top surface, the 3D design places only the hot junctions on the top plane while all cold junctions are on the bottom plane. Since the top surface is only covered with hot junctions, the whole top surface could be utilized as sensing area for temperature monitoring, which is much larger than the in-plane thermopile layout. Furthermore, free standing membranes could be avoided in the 3D design, thus making the device more robust during operation. For 3D layout, usually thermal legs over the vertical direction to thickness of 20 μm and above were grown on silicon or polyimide (PI) substrates using electroplating methods [16,17]. Recently, thermopiles consisting of 64,000 to 147,000 p/n silicon bridge-type thermocouples in one centimeter square were fabricated [18]. The design includes vertical p/n silicon thermal legs connected by Cr/Au metals which act as the hot and cold junction of the thermocouple. The thermocouples have the free-standing bridge structures with 2.5 μm air gap to the base silicon wafer. However, electroplating method is not as commonly available as the thin film deposition method in many IC fabrication facilities. To avoid electroplating, n/p silicon wafers were anisotropically etched to form thermal legs and joined together layer-by-layer to make the 3D structure [19]. Alternatively, PI substrate with slopped valleys was etched using alkaline solution to form thermocouples by thin film deposition instead of vertical through holes for electroplating [20].

With the development of technology, high throughput applications become more and more important and demanding, for example, the development of enthalpy arrays using thermistors as heat detectors for high throughput applications [21,22]. However, many thermopile-based microcalorimeters are not designed for high throughput applications, *i.e.*, they have only one signal output channel. The sensing area of current chip calorimeters is usually small, which is not suitable for many applications, for example, the next generation biofilm reactor. In this paper, we have developed a silicon-based 3D multichannel chip calorimeter, which is IC compatible without the dependence on an electroplating facility, *i.e.*, it is convenient and powerful for packing thermocouples with high density. Furthermore, it has a large sensing area (20 mm × 20 mm), and more importantly, the large sensing area could be divided into small sensing units for applications requiring high throughputs or high spatial resolution. Suggestions were also provided to further improve the performance, for example, to select materials with better thermoelectric effect [23–25].

## 2. Design and Fabrication of the Thermopile

**Figure 1.**
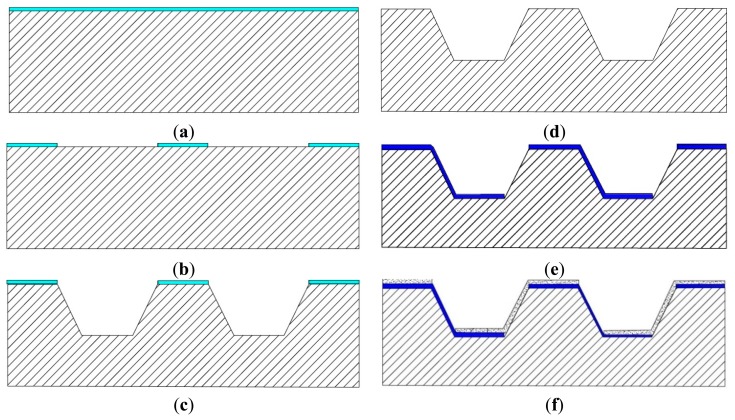
Fabrication process of the thermocouple array. (**a**) Oxidized Silicon wafer; (**b**) Patterning oxide to form etching windows; (**c**) TMAH anisotropic etching; (**d**) Remove oxide to form the template; (**e**) Depostion and patterning Ni; (**f**) Deposition and patterning Au.

The designed chip calorimeter is composed of Ni/Au thermocouples connected in series on an alkaline etched Si (100) substrate. The fabrication process started with wet oxidation of a 4-inch silicon wafer (100) to grow 500 nm oxide as an etching mask (Figure 1a). Shipley S1813 photoresist and its developer were used in all photolithography steps of the fabrication process. The thermal oxide film is patterned by photolithography using a Karl-Suss MA6 mask aligner and the buffered oxide etching (BOE, HF:NH_4_^+^ = 5:1) solution to form etching windows (Figure 1b). The silicon wafer was etched in tetramethylammonium hydroxide (TMAH) 25% at 80 °C to form cavities (Figure 1c). The square cavity has the dimension of 200 μm with 7 μm depth and 100 μm spacing. Due to crystallographic properties of silicon(100), alkaline etching will form a cavity with sloped side walls with an angle of 54.7°, which enables the deposition and coverage of the thermoelectric materials in the later steps. The top and bottom surfaces of the cavity are where the thermocouple forms the hot and cold junctions respectively (Figure 1). The thermal oxide film is then removed by dipping in buffered oxide etch (BOE) solution for 15 min (Figure 1d). After extensive wafer cleaning and drying, Ni thermocouple legs with 400 nm thickness were deposited by electron beam evaporation and patterned by lift-off process (Figure 1e). Similarly, 400 nm Au thermocouple legs and wire bonding pads were formed by sputtering and lift-off process (Figure 1f). Each thermal leg is 180 μm long, 50 μm wide, 30 μm overlapped with the other, and the spacing between thermal legs is 250 μm. Overall, there are 4489 thermocouples distributed over the chip area of 20 mm × 20 mm. The thermocouple density per area is 11 mm^−2^ in the current design, which can be easily increased with smaller feature size. The fabricated chip was then glued on a printed circuit board (PCB) and wire bonding was carried out to form the connection between the chip bond pads and the PCB metal pads. The PCB was designed to have through holes filled with copper to ensure good thermal contact between the cold junctions and the heat sink. This design creates a 3D structure thermopile where the hot and cold junctions are no longer on the same plane but two parallel planes, which is different from the conventional in-plane layout and enables the whole chip area now to serve as the sensing area. Furthermore, these thermopiles are divided into smaller sensing areas for applications that require multichannel, high throughputs or high spatial resolution.

## 3. Device Characterization

### 3.1. Microcopy

The thermocouple elements on the chip were investigated by scanning electron microscope (SEM, Hitachi 3500N, Tokyo, Japan) to confirm the 3D structure of the standing thermopile with the hot and cold junctions.

### 3.2. Temperature Sensitivity

**Figure 2.**
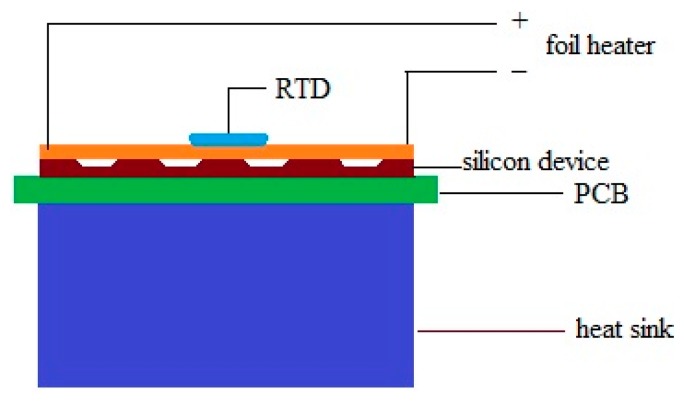
Joule heating experiment illustration.

Performance of the device was evaluated by a Joule heating experiment (Figure 2) [18–20]. A foil heater powered by a DC source was attached on top of the device which was cover by a glass cover slip with 170 μm thickness.

The temperature of the heater is monitored by a resistance temperature detector (RTD) thermometer (SA1-RTD-4W, Omega Engineering, Stamford, CT, USA). The whole system was placed inside an environment chamber with pre-setting temperature of 25 °C to minimize the temperature fluctuation of the surrounding environment. The temperature of the heater was firstly raised up to 49.6 °C and cooled down step by step to achieve the output signal at different temperatures. Sufficient time was allowed at each temperature point so that the system can be stabilized to steady state.

### 3.3. Heat Power Sensitivity

**Figure 3.**
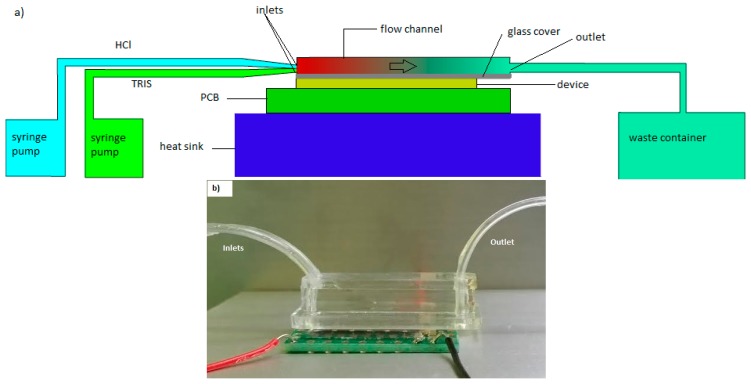
(**a**) Illustration of the flow reaction experiment; (**b**) Flow chamber on top of the chip calorimeter.

To demonstrate the calorimetric application, a flow reaction experiment was carried out. Polydimethylsiloxane (PDMS) was used to fabricate the flow chamber with a width of 4 mm, height of 1.5 mm and the effective length on the chip is 20 mm. The bottom of the flow channel was covered by a thin microscope cover glass with a thickness of 170 μm and attached to the thermopile device. Two inlets were designed to allow the TRIS and HCl mixing to happen right after they enter the channel (Figure 3).

Tris(hydroxymethyl)aminomethane (TRIS, 0.1M) and hydrochloric acid (HCl, 0.0lM) were injected separately by a syringe pump at different flow rates via two inlets. The TRIS concentration was used 10 times higher than HCl to ensure that the reaction is mostly completed. The whole set up was placed in an environmental chamber to minimize temperature disturbance. The injections were controlled by a computer. The bottom of the PCB is attached on an aluminum block acted as a heat sink with water bath underneath to remove the heat regenerated. The output voltage was read by a nanovoltmeter (34420A, Agilent, Santa Clara, CA, USA) and recorded by computer using the LabView program.

## 4. Results and Discussion

**Figure 4.**
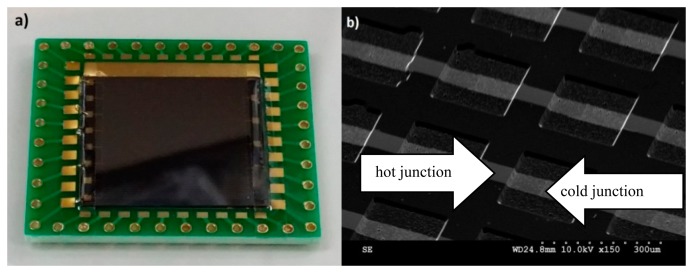
(**a**) Optical picture of the chip calorimeter bonded on a PCB; (**b**) SEM image of the hot and cold junctions of the thermopiles.

The microfabricated chip calorimeter is bonded on a PCB board for all experiments (Figure 4a). The SEM image confirms the micro-structure of the fabricated thermocouple array (Figure 4b). The deposited thin metal films cover the sloped sidewalls and the edge properly. The two metals are overlapped on the top of the ridges and at the bottom of the valleys to form hot junctions and cold junctions, respectively.

**Figure 5.**
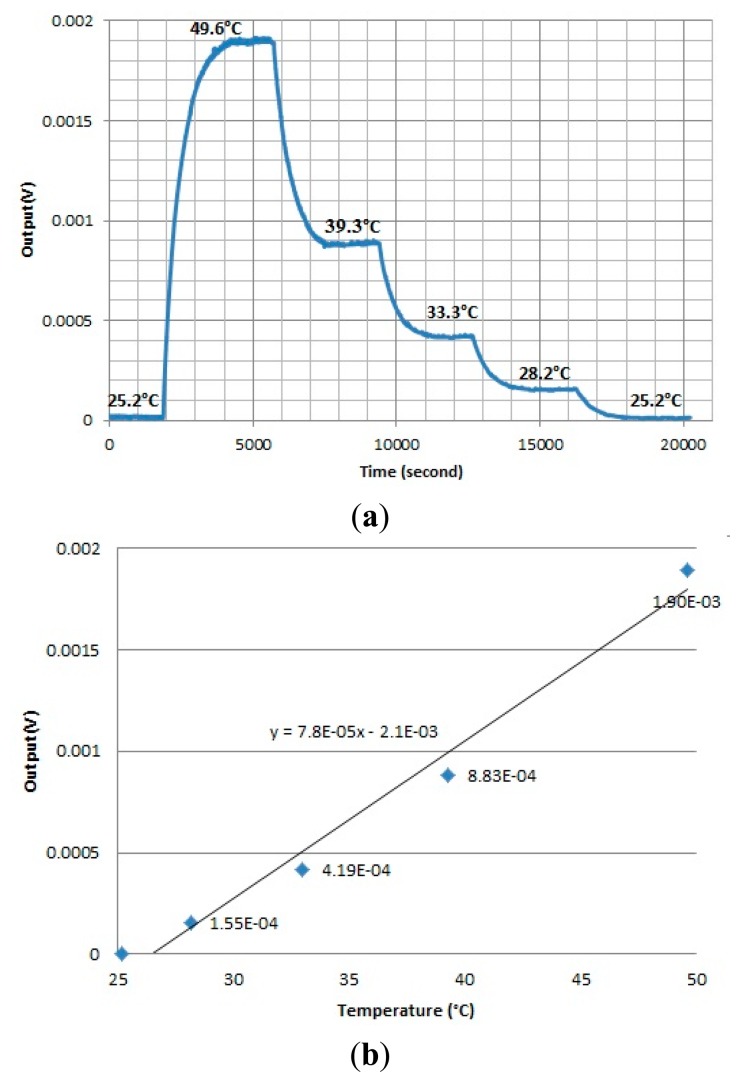
(**a**) Responses of the chip calorimeter towards Joule heating; (**b**) Output voltage *vs.* temperature plot.

The output signals from the chip calorimeter were plotted at temperature differences generated by the foil heater (Figure 5a). When the temperature was decreased by reducing the voltage supplied to the heater, the output signal dropped accordingly. The average output signal at different temperatures was plotted against the temperature (Figure 5b). The slope of the regression line was estimated as the temperature sensitivity of the device (78 μV/K).

**Figure 6.**
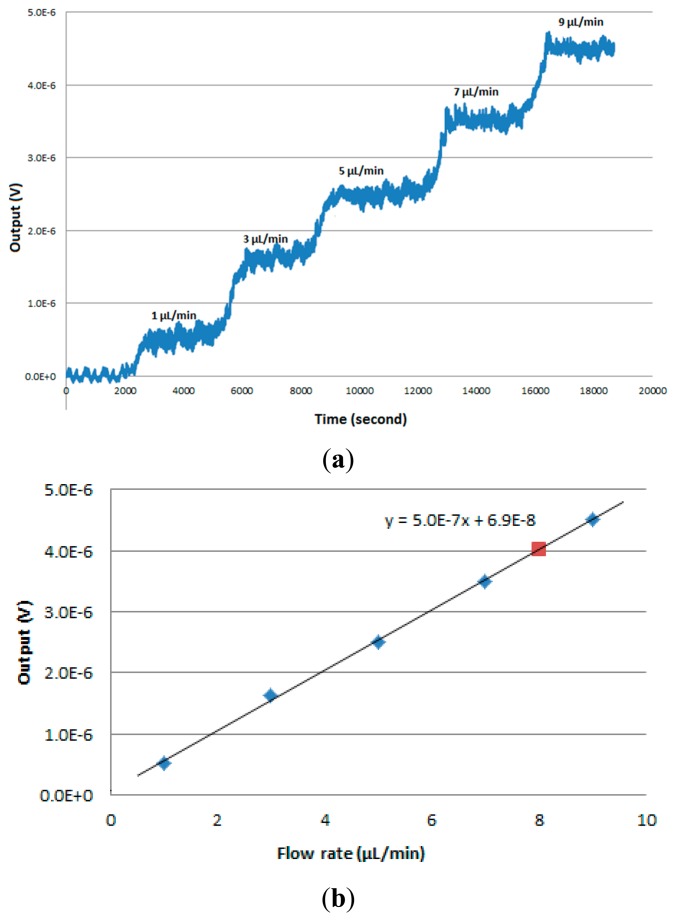
(**a**) Output signal at different flow rates in TRIS/HCl reaction; (**b**) Output signal *vs.* flow rate.

For the flow reaction experiment, the flow rate started at 1 μL/min and was increased to 3, 5, 7 and 9 μL/min, respectively. The output signal at different flow rates were collected (Figure 6a). The baseline fluctuation when the flow rate is zero was estimated by the standard deviation at 50 nV. The average signal outputs at different flow rates were plotted against the flow rate (Figure 6b). The slope of the regression line was estimated as *s* = 0.5 μV·min·μL^−1^. The enthalpy (heat) of protonation of TRIS is −47.4 kJ/mol [26]. The heat power sensitivity *S* was calculated using the equation below:

(1) $$S=\frac{s}{C_{\text{HCl}}\times Q_{\text{reaction}}}=\frac{0.5\times 60}{0.01\times 47400}=63.2mV/W$$

where *s* is the slope of the regression line, *C_HCl_* is the concentration of HCl, *Q_reaction_* is the enthalpy of protonation.

The estimated temperature sensitivity and heat power sensitivity are not as high as some previous reports [15,27,28], but are good enough for some applications in drug screening, biofilm culture, *etc.* If needed, the sensitivities could be improved using high performance materials such as bismuth, antimony, telluride alloys or silicon (above 100 μV/K) [26]. Furthermore, in the current experimental design, a standard glass cover slip is used to isolate the chip calorimeter from the reaction chamber because it is frequently used in biological applications, for example, biofilm culture. The sensitivity and time response of the chip calorimeter could be further improved by replacing the glass cover slip with thinner membranes [3,12] to provide the necessary isolation and better heat transfer between the fluid in the reaction chamber and the thermopile device.

**Figure 7.**
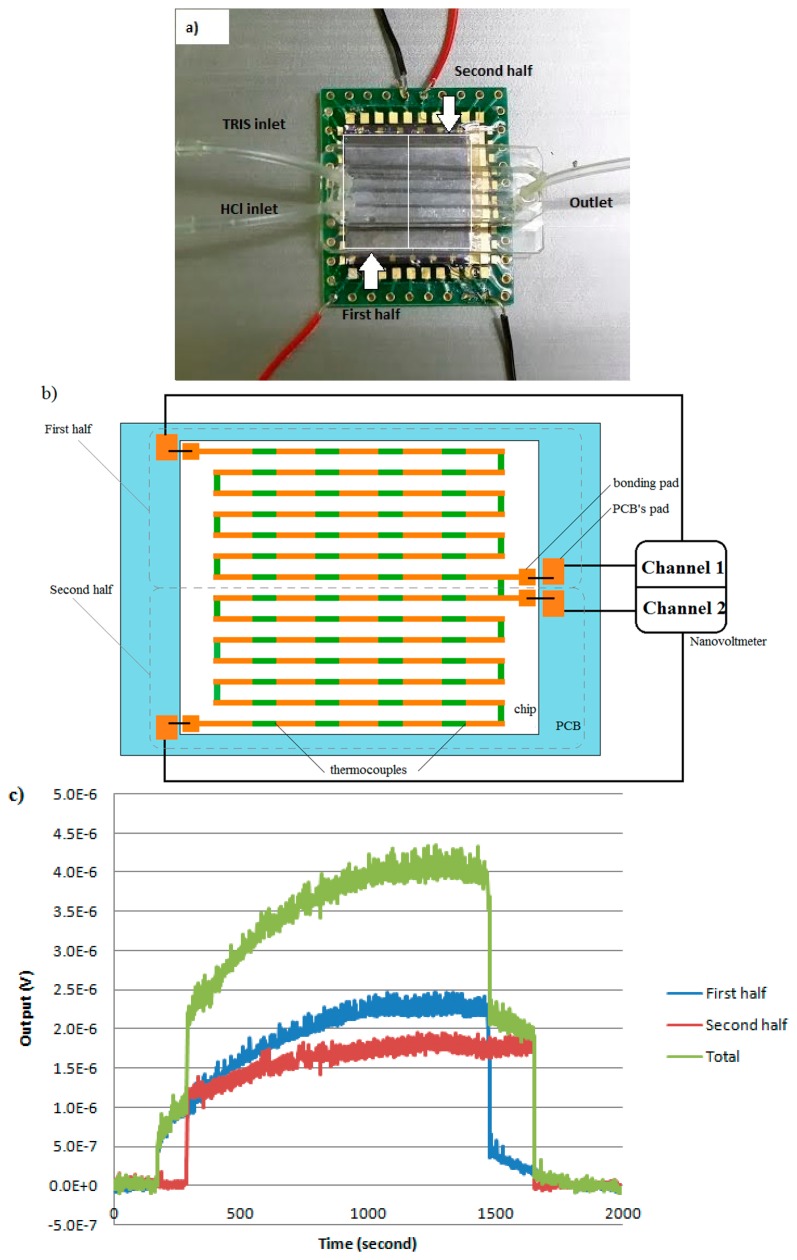
(**a**) Device is divided into 2 halves, each of them connected to an independent readout channel. (**b**) Illustration of the area-partitioning mechanism. (**c**) Data collected from the two channels.

The current design also enables multiple channel outputs from one chip calorimeter for applications requiring high throughput or high spatial resolution of heat generation, such as drug screening, or biofilm monitoring. As an example, two isolated and independent signal outputs from one chip calorimeter has been successfully collected, where the single chip device was divided into two parts (Figure 7a) and recorded simultaneously by appropriate design of the readout circuit. Figure 7b illustrates the partitioning mechanism together with the layout of Ni/Au thermocouples. Bonding pads on the chip calorimeter are connected to a PCB to enable convenient and flexible connection to the reading instrumentation in a real application. Particularly in this experiment, a nanovoltmeter (Agilent 34420A) with two independent channels was connected with the output terminals on the PCB to read signals from the first half and second half of the chip. The measurement was made alternatively for each channel every three seconds. The same experimental conditions as the heat power sensitivity experiment were used with the flow rate of 8 μL/min. As shown in Figure 7c, the output signals started and stopped immediately after turning on and off the flow, which means that the reaction actually started immediately when mixing at the inlet. The delay time for the second half signal as compared to the first half is due to the low flow rate. Overall, both signals reached stabilized stage and the first half of the flow cell (close to the inlet) has generated more heat compared to the second half (close to the outlet). The stabilized total output signal under the 8 μL/min flow rate is around 4 μV, which fits well in the linear regression plot (Figure 6b).

## 5. Conclusions

We have successfully developed a chip calorimeter with a large sensing area and multichannel capacity based on a 3D thermopile layout, which is highly desirable for many applications requiring large sensing area or high throughputs, such as biofilm research, or drug screening. The device performance features, such as temperature sensitivity and heat power sensitivity, have been characterized and potential improvements have been identified for future researches.
